# Digital Adoption by an Organization Supporting Informal Caregivers During COVID-19 Pandemic Showing Impact on Service Use, Organizational Performance, and Carers’ Well-Being: Retrospective Population-Based Database Study With Embedded User Survey

**DOI:** 10.2196/46414

**Published:** 2024-05-13

**Authors:** Ala Szczepura, Amir Jahan Khan, Deidre Wild, Sara Nelson, Sonja Woodhouse, Mark Collinson

**Affiliations:** 1 Research Centre for Healthcare & Communities Coventry University Coventry United Kingdom; 2 Department of Economics Institute of Business Administration (IBA) Karachi Pakistan; 3 Prostate Cancer Research London United Kingdom; 4 Carers Trust UK London United Kingdom; 5 MC2S Consultancy Services Bromsgrove United Kingdom

**Keywords:** digital adoption, COVID-19, informal caregivers, carer support organization, organization performance, integrated care systems, care systems, health policy, aging in place, digital divide

## Abstract

**Background:**

The COVID-19 pandemic has catalyzed a move from face-to-face to digital delivery of services by hospitals and primary care. However, little is known about the impact of digital transformation on organizations supporting unpaid caregivers. Since the start of the COVID-19 pandemic, the value of care provided by such informal caregivers is estimated to be £111 billion (US$ 152.7 billion) in England.

**Objective:**

This study aims to analyze service uptake patterns (including digital service options) over the pandemic period in an English caregivers’ support organization covering a population of 0.98 million; measure changes in organizational performance, service efficiency, and quality; and identify the views of caregivers on service provision and future digital delivery.

**Methods:**

This was a retrospective analysis of the use of digital versus nondigital support services (January 2019 to June 2021) by caregivers in city and rural geographic areas. We compared organizational performance and service quality indicators for 2 financial years (2019-2020 and 2020-2021). A survey was conducted to identify barriers and facilitators to digital service uptake, the computer proficiency of caregivers (the Computer Proficiency Questionnaire, 12-item version), and preferences for future digital service provision. Quantitative data were analyzed using Stata 13 (StataCorp LLC). Thematic analysis was used for open-text survey responses.

**Results:**

The number of caregivers registered with the organization rose from 14,817 in 2019 to 20,237 in 2021. Monthly contacts rose from 1929 to 6741, with remote contacts increasing from 48.89% (943/1929) to 86.68% (5843/6741); distinctive patterns were observed for city versus rural caregivers. There was an increase in one-to-one contacts (88.8%) and caregiver assessments (20.9%), with no expansion in staffing. Service quality indicators showed an improvement in 5 of 8 variables (all *P*<.05). The 152 carers completing the survey had similar demographics to all registered caregivers. The Computer Proficiency Questionnaire, 12-item version, mean score of 25.61 (SD 4.40) indicated relatively high computer proficiency. The analysis of open-text responses identified a preference for the organization to continue to offer face-to-face services as well as web-based options. The digital services that were the most highly rated were carers’ well-being assessments, support needs checks, and peer support groups.

**Conclusions:**

Our findings show that staff in the caregiver support organization were agile in adapting their services to digital delivery while dealing with increased numbers of registered clients and higher monthly contacts, all without obvious detriment to service quality. Caregivers indicated a preference for blended services, even while recording high computer proficiency. Considering the economic importance of unpaid caregivers, more attention should be given to organizations funded to provide support for them and to the potential for technology to enhance caregivers’ access to, and engagement with, such services.

## Introduction

### Background

During the COVID-19 pandemic, social distancing policies limiting physical contact transformed how people were able to access health and care services and sped up digital transformation in many sectors. In the United Kingdom, traditional hospital services rapidly moved from face-to-face to digital services, from simple websites to web-based platforms, to reduce the chance of infection transmission [1]. In primary care, general practitioners (GPs) also adopted a *digital first* approach for consultations [2]. However, how those organizations that are contracted to support unpaid caregivers adapted their services is not known [3]. Such organizations are not part of the National Health Service (NHS) but are instead contracted by local government [4]. Before the pandemic, expenditure on the services these organizations provided was reduced by 11% over the 6-year period from 2015 to 2021 [5], while 36,000 more carers were directed to their services [5]. It is recognized that unpaid carers play a crucial role in providing essential care worldwide [6]. Bearing these facts in mind, the absence of research on how these organizations moved to web-based delivery, what impact this had on service delivery, their ability to do this without affecting service quality, and the response of client caregivers are noteworthy.

Before the pandemic, an NHS report on the *Widening Digital Participation Programme* identified that organizations supporting the well-being of carers had largely been forgotten [7]. Currently, many countries are introducing policies of *active aging*, supporting older people to live independently in the community rather than entering expensive long-term residential care [8,9]. As a result, while the number of staff employed by care homes has remained unchanged from 2012 to 2022 in England, there has been a 27% increase in domiciliary care workers who support people in their own homes [10]. The pressures on unpaid carers have inevitably increased. Currently, the care provided by unpaid carers in England is estimated to be equivalent to that provided by 3.2 million full-time paid care workers [11,12], nearly 6 times the size of the paid workforce of 510,000 domiciliary care workers [13]. In addition, the continuity of care provided by family carers is becoming increasingly important in the context of a high annual turnover of domiciliary care staff (31.5% in England) [13].

Across the world, countries are developing integrated long-term care strategies to support their aging populations as recommended by the World Health Organization [14]. England has established 42 new integrated care systems (ICSs) to underpin integration between health and care services in these geographic areas [15]. Proposals for joining up care include the idea of *wrap-around services* for care recipients plus their caregivers, but there is no mention of organizations that provide support for unpaid caregivers. Although the national ICS strategy incorporated a digital plan, this currently excludes mention of organizations that support informal carers [16]. Such services are typically provided by charities or not-for-profit organizations that themselves may have limited expertise in digital transformation [17]. In this context, it is important to better understand the experiences of such carer support organizations during the pandemic and the response of the caregivers they support.

### Objectives

This study aims to provide evidence to address this important research gap. The research has 3 main objectives: to analyze changes in service use patterns (including services accessed and the uptake of digital options); to assess any impact on organizational performance, service efficiency, and key quality indicators; and to identify user clients’ preference for future digital services.

## Methods

### Overview

We analyzed data collected by an organization providing support for 20,237 caregivers, covering city and rural geographic areas. Data were downloaded and fully anonymized. The uptake of digital and traditional services was examined over a 30-month period from January 2019 (before the pandemic) to June 2021 (after COVID-19–related restrictions were lifted). Service-level performance and proxy quality indicators were constructed and compared for the prepandemic financial year (2019-2020) and through the following initial lockdowns (2020-2021). The analysis of a user feedback survey undertaken at the end of this period (September 2021) explored barriers and facilitators to digital service uptake, the computer proficiency of caregivers, and views on future digital service provision. The organization’s digital preparedness before the pandemic was assessed.

### Ethical Considerations

This retrospective study using fully anonymized existing data received ethics approval from the Coventry University Ethics service (P163079).

### Organizational Setting

The study was undertaken at the Carers Trust Heart of England (CTHE). The organization operates in the complex UK sector that provides support for unpaid carers [4]. The CTHE is contracted to provide carer assessments and caregiver well-being services in 2 separate geographic areas: city area (Coventry) and rural area (Warwickshire, including towns and villages). The 2 areas have a total population of 0.98 million, and they are covered by a single ICS. Services provided include an assessment of a caregiver’s needs, information on health and care services, benefit entitlement, assistive technologies, and peer support. Before the pandemic, the CTHE had made a number of changes to its IT systems, moving everything to a single cloud platform so that databases could be accessed from anywhere. Laptop computers and mobile phones had been provided to all frontline staff, all of which greatly facilitated home working. Job descriptions were also adapted to mention hybrid working. Throughout the observation period, the number of CTHE staff providing direct support to adult carers remained relatively constant at 14 well-being advisers, 3 administrators, 3 specialist roles (ethnic minority support worker, mental health worker, and carer trainer), and a manager for each area. A separate team provided support for young carers; this activity was excluded from our study. The CTHE is a member of a network of 124 Carers Trust partners across England, Scotland, and Wales. Members work within a national framework of policies, procedures, and internal quality assurance programs.

### Longitudinal Data and Analysis

#### Overview

CTHE staff downloaded select activity data routinely collected for adult carers registered with the service (Multimedia Appendix 1). Young carers (aged <18 y) were excluded. Data were fully anonymized before being provided to the research team for analysis. Data cleaning and analysis were conducted on the imported raw data using reproducible coding files. Statistical analysis used Stata 13 (StataCorp LLC). The hypothesis was that there would be an increase in the use of web-based methods, although it was unclear whether digital levels would be sustained, what the impact on service delivery levels and quality would be, or what views client carers might have on a future digital service.

#### Service Use Patterns

Descriptive statistics were used to examine service use patterns and changes in the means of accessing services (ie, in person vs digital) [18]. Monthly contacts handled by the service were the primary variable used to explore use patterns over the 30-month period (January 2019 to June 2021). This covered two 15-month pre- and postpandemic periods, with the first national lockdown occurring midway in March 2020. The levels of service use by city versus rural carers were examined, together with the primary reasons for contacting the CTHE.

#### Service Performance Levels and Quality Indicators

Four key activity measures reported to commissioners each financial year were extracted from the data downloads. These included the number of carers supported, the number of one-to-one contacts, the number of carers’ assessments completed, and numbers of carers attending group activities. The CTHE also collected structured feedback from the client after every contact (Multimedia Appendix 1). Five proxy quality indicators routinely reported to the funder were also extracted. A further 3 proxy quality indicators were constructed from the raw data to identify whether a contact had *reduced stress*, *increased control of personal life*, or *increased confidence*. Changes in all 8 quality indicators were compared for the 2 financial years 2019-2020 and 2020-2021, with *P* values for percentage changes estimated using the Pearson chi-square test of association between the characteristic variables and the corresponding totals [19].

### Survey of Client Caregivers

Registered adult carers were invited by the CTHE to complete a feedback questionnaire in September 2021 to review provision and help plan future services (Multimedia Appendix 2). Responses were fully anonymized before being provided to the research team, with a minimum target of 60 responses set for analysis. Closed questions requested information on the use of digital services, barriers and facilitators to access, and satisfaction with the services accessed, while open-text comment boxes enabled people to expand on their responses. Respondents were also invited to complete the Computer Proficiency Questionnaire, 12-item version (CPQ-12) to assess their computer proficiency [20]. As well as being made available on the web, feedback questionnaires were also mailed by the CTHE to carers. The characteristics of respondents were compared to those of adult carers on the register where possible. Open-text responses were analyzed for content using thematic analysis and cross-referenced to gain an understanding of the underlying reasoning behind the views expressed [21].

## Results

### Longitudinal Data Analysis

#### Registered Carer Characteristics

Data on registered carers (Multimedia Appendix 1) were analyzed over the 30-month period from January 2019 to July 2021. During this time, the total number of carers registered with the organization increased from 14,817 to 20,237 (Table 1). Those living in the rural area rose from 1685 to 4778 (183.6% increase), while those in the city showed a more modest rise from 13,132 to 15,459 (17.7% increase). Overall, the proportion of rural caregivers increased from 1 in 10 (1685/14,817, 11.37%) to 1 in 4 (4778/20,237, 23.61%).

**Table 1:** Demographics of registered caregivers and survey respondents.

Characteristics			Caregivers, n (%)
**Rural caregivers**			
	January 2019^a^		1685 (11.37)
	January 2020^b^		3035 (17.63)
	July 2021^c^		4778 (23.61)
**Registered caregivers (n=17,641; March 2020)^d^**			
	**Race and ethnicity**		
		Black (Caribbean or African)	406 (2.3)
		South Asian	2382 (13.5)
		White	13,972 (79.2)
	Sex (female)		11,467 (65)
	Aged ≥65 years		9067 (51.4)
	**Employment status**		
		Retired or gave up work to care	7868 (44.59)
		Working or in training	3369 (19.1)
		Unemployed	1270 (7.2)
**Survey respondents (n=152; September 2021)**			
	Rural caregivers^e^		80 (56.3)
	Sex (female)		97 (63.8)
	**Employment status**		
		Retired or gave up work to care^f^	33 (59)
		Working or in training^f^	18 (32)
		Unemployed^f^	2 (3)

^a^Total registered carers: 14,817.

^b^Total registered carers: 17,246.

^c^Total registered carers: 20,237.

^d^Midway through the study period.

^e^142 respondents provided information on their location.

^f^56 respondents provided information on their employment status.

The demographic characteristics of registered carers were analyzed based on data recorded midway through the observation period from January 2019 to March 2020 (Table 1). The age and sex breakdown is comparable to national figures for carers [12]. Two-thirds (11,467/17,641, 65%) of the respondents were female carers; approximately half (9067/17,641, 51.4%) were aged ≥65 years; and over three-quarters (13,972/17,641, 79.2%) were White. In terms of their employment status, nearly half (7868/17,641, 44.59%), the largest group, were retired or had given up work to care, with only 1 in every 5 (3369/17,641, 19.1%) working or in training.

#### Service Use Patterns

**Figure 1:**
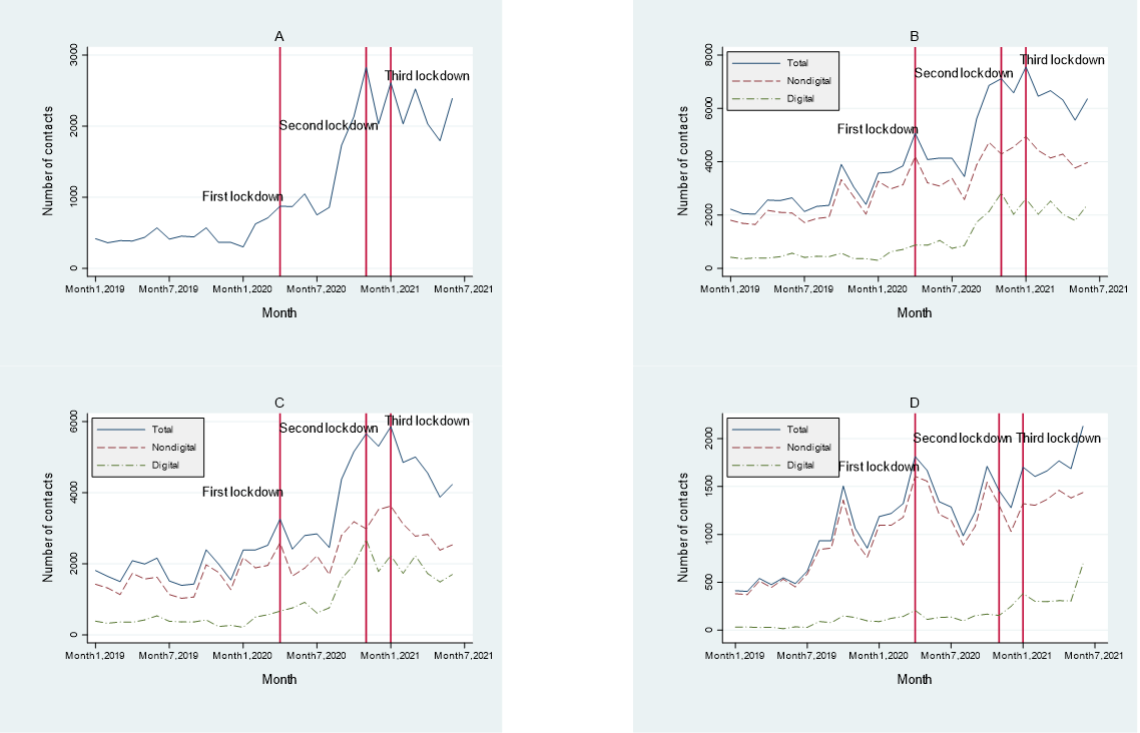
Number and types of contacts. (A) Digital contacts (all carers). (B) Breakdown of digital and nondigital contacts (all carers). (C) City carers (breakdown of digital and nondigital contacts). (D) Rural carers (breakdown of digital and nondigital contacts).

Over the 30-month period (January 2019 to July 2021), digital services were provided alongside face-to-face services. The former included the use of email, SMS text messaging, Zoom, WhatsApp, and Microsoft Teams, as well as web-based group sessions. Use patterns were analyzed over time (Figure 1). The 3 vertical lines indicate the time points at which major national COVID-19–related restrictions were applied (ie, lockdown periods). From 2019 to 2021, monthly carer contacts with the well-being support service rose from 1929 to 6741, with telephone contacts rising from 818 to 3071 per month, and digital contacts from 125 to 2772 per month. A separate analysis of digital contacts during this period (Figure 1A) shows that, alongside a near–5-fold overall increase in the monthly rate, there were peaks coinciding with the national lockdowns. A separate analysis of digital versus nondigital contacts (Figure 1B) uncovers a clear change in the balance between the two, with digital contacts rising from 6.48% (125/1929) to 41.12% (2772/6741) of all contacts by the end of the period.

A separate examination of rural and city caregivers uncovers distinctly different patterns. For city carers (Figure 1C), digital contacts started to climb steadily from the start of the pandemic, almost reaching parity with face-to-face contacts around the time of the second lockdown. They then started to tail off toward the end of the 30-month observation period. Rural carers (Figure 1D) demonstrated a much slower initial uptake, with the rate of digital adoption only really starting to pick up after the second and third lockdowns. However, unlike in the case of city carers, rates were continuing to rise at the end of the observation period.

#### Types of Digital Contacts Used

**Figure 2:**
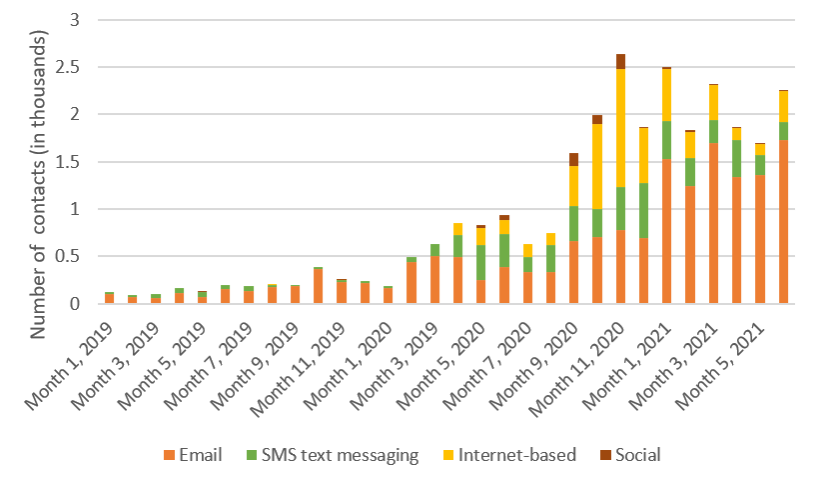
Number of digital contacts per month versus type of remote access.

Digital contacts were categorized into 4 broad groups. Two represented more flexible asynchronous methods (ie, email and SMS text messaging), whereas 2 represented fixed-time synchronous methods (ie, internet-based communication using Zoom, WhatsApp, and Microsoft Teams) and social digital group activities. Before the pandemic, only asynchronous methods were used, and rates were very low (Figure 2). With the first national lockdown (month 15), the use of other digital methods started to be added. Over time, the use of internet-based communication increased, overtaking SMS text messaging, although email remained the principal form of contact. “Social” groups were the least used form of digital contact used, possibly due to greater difficulties in arranging and delivering these. Their use peaked between September 2020 and January 2021 (ie, between the second and third lockdowns).

#### Types of Services Accessed

Well-being services available to carers at the start of the pandemic were mostly delivered via carers center appointments, home visits, and various outreach activities. During the pandemic, carers center appointments ceased after the first lockdown, with home visits and outreach activities significantly reduced. The well-being services provided were information and advice on aids and adaptations, carers’ assessment, education and training (including digital), emotional support, local and national health and social care services, personal care and health, residential care and day care (for cared-for persons), and social inclusion and interests (including links to local and national groups). Other services included peer support and emergency planning. During the pandemic, additional services were introduced to address specific needs: a *grief and loss* service, *employment support* working collaboratively with employers, and *finance support* to reduce carer hardship.

**Figure 3:**
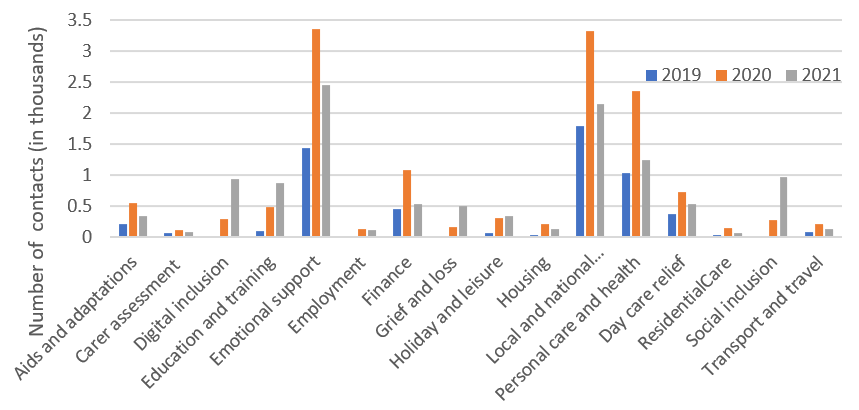
Pattern of carer support services contacted over time.

Analysis of the reasons for contacting the well-being service over the 3-year period from 2019 to 2021 indicated that, irrespective of the year, the 3 most common reasons were *emotional support*, *local and national information*, and *personal care and health* (Figure 3). All 3 reasons peaked in 2020 (early pandemic period), as did information on *day care relief* and *aids and adaptations*. Advice related to *digital inclusion* and *social inclusion* were unusual in showing a steep rise in the final 6 months, from a base where both were 0 in 2019. *Education and training* showed a similar pattern, possibly partly related to digital training needs. The most frequently new service accessed was *finance*, with the *grief and loss* service reaching a similar level in the final 6 months.

### Service Performance and Quality

Activity levels demonstrated an increase of 88.8% over 2 years in the numbers of one-to-one contacts and an increase of 20.9% in individual carer assessments (Table 2). At the same time, there was a decrease of 70.6% in numbers attending group activities. Among the 5 proxy quality indicators routinely reported to the funder, those critical to well-being all showed an improvement (ie, *feeling less alone*, *making it easier to cope with caring role*, and *helping improve physical health*), as did the extra 3 indicators constructed (ie, *reduced stress*, *increased control of personal life*, and *increased confidence*). Improvements ranged from 13% for reduced stress (*P*=.001) to 1% for others (*P*>.05). The only measure to show a significant decrease was *help dealing with health and social care professionals* (*P*=.001). This may, in part, be due to pressures experienced by health and social care staff during the pandemic.

**Table 2:** Change in carer contact type and quality indicators (from 2019-2020 to 2020-2021).

			2019-2020	2020-2021	Change (%)	*P* value^a^
**Carer contact type**						
	One-to-one contacts with carers, n		7344	13,868	+88.8	<.001
	Carers supported on one-to-one basis, n		3021	4463	+47.7	<.001
	Carer assessments completed, n		234	283	+20.9	.03
	Carers attending group activities, n		670	197	−70.6	<.001
Registered caregivers, n			14,817^b^	17,641^c^	+19.1	N/A^d^
**Benefits reported after contact**						
	**Helped me feel less alone in my caring role**					
		Contacts, n	2249	5217	+131.0	<.001
		Contacts (%)	33	36	+9.0	N/A
		Caregivers expressing this, n	1134	2410	+112.5	<.001
	**Helped me reduce my stress**					
		Contacts, n	1771	4378	+147.2	<.001
		Contacts (%)	18	31	+72.2	N/A
		Caregivers expressing this, n	948	2410	+154.2	<.001
	**Made it easier to cope with my caring role**					
		Contacts, n	825	1319	+59.9	<.001
		Contacts (%)	8	9	+12.5	N/A
		Caregivers expressing this, n	597	879	+47.2	.95
	**Helped me improve my physical health**					
		Contacts, n	465	1278	+174.8	<.001
		Contacts (%)	5	9	+80.0	N/A
		Caregivers expressing this, n	293	660	+125.3	<.001
	**Helped me improve my financial position**					
		Contacts, n	692	804	+16.2	<.001
		Contacts (%)	7	6	−14.3	N/A
		Caregivers expressing this, n	469	513	+9.4	<.001
	**Helped me deal with health and social care professionals**					
		Contacts, n	342	250	−26.9	<.001
		Contacts (%)	3	2	−33.3	N/A
		Caregivers expressing this, n	230	174	−24.3	<.001
	**Helped me increase control of my personal life**					
		Contacts, n	305	638	+109.2	.15
		Contacts (%)	3	4	+33.3	N/A
		Caregivers expressing this, n	218	350	+60.6	.35
	**Helped me increase my confidence**					
		Contacts, n	205	417	+103.4	.39
		Contacts (%)	2	3	+50.0	N/A
		Caregivers expressing this, n	144	272	+89.9	.02

^a^Pearson chi-square test.

^b^January 2019.

^c^March 2020.

^d^N/A: not applicable.

### Survey of Client Caregivers

#### Respondents

A total of 152 caregivers completed the survey. Personal characteristics (age and sex) were largely comparable to those of all caregivers registered with the CTHE (Table 1). Ethnicity was not recorded in the survey. In terms of employment, the 2 largest groups were those who had retired or people who had given up work to care and those in work or training. The survey respondents included a higher percentage of rural caregivers than the carer register (85/142, 59.9% vs 4778/20,237, 23.61%); of the 152 respondents, 10 (6.6%) did not provide information on their location. Information provided in the survey showed that nearly half of these respondents (70/152, 46.1%) were caring for a husband, wife, or partner and 27.6% (42/152) for a parent, whereas the remaining 26.3% (40/152) had *another relationship*. Nearly one-third (41/148, 27.7%) of people providing more detailed information were caring for someone who was living on their own, and in 78.9% (112/142) of responses, care was provided solely by the unpaid caregiver. Where there was access to support from domiciliary carers, this averaged 19.5 (SD 30.7; range 2-40) hours per week. The CPQ-12 Questionnaire was completed by 94 (61.8%) of the 152 respondents. Respondents achieved a mean score of 25.61 (SD 4.40), with city and rural carers exhibiting similar proficiencies (Multimedia Appendix 3 [20,22]).

#### Prepandemic Use of Services and Main Barriers

Historically, respondents reported using a broad range of services (Table 3). Reasons for not accessing a particular service were provided by 82.2% (125/152) of the respondents; the remainder (27/152, 17.8%) reported only registering with the CTHE after the first lockdown. Two barriers to use were timing and travel distances, but far more respondents, from 28.6% (30/105) to 39.6% (40/101), perceived lack of awareness of a service as a barrier to its use. A further 33% (30/90) to 44.6% (45/101) stated that, before the pandemic, they had no need for a particular service. During the pandemic, some respondents had stopped using community outreach sites (14/114, 12.3%) and carers centers (19/114, 16.7%); a very small percentage (from 2/114, 1.8% to 4/114, 3.5%) had started to use these services. With cessation of home visits, 21.9% (25/114) reported that they had started to use telephone support during the pandemic.

**Table 3:** Carers’ use of services before the pandemic and perceived barriers to use.

Service	Used 1-12 times (2019), n/N (%)	Barriers to use of a service, n/N (%)			
		Timing not suitable	Too far to travel	Not aware of service	Not needed
Carers center (drop-in visit)	23/116 (19.8)	12/105 (11.4)	5/105 (4.8)	30/105 (28.6)	37/105 (35.2)
Carers center (appointment visit)	16/117 (13.7)	8/101 (7.9)	5/101 (5)	32/101 (31.7)	42/101 (41.6)
Home visits	16/112 (14.3)	9/101 (8.9)	2/101 (2)	30/101 (29.7)	45/101 (44.6)
Outreach (eg, community site)	14/114 (12.3)	4/101 (4)	5/101 (5)	40/101 (39.6)	36/101 (35.6)
Telephone support	48/122 (39.3)	4/88 (4.5)	0/88 (0)	28/88 (31.8)	34/88 (38.6)
Group activities	34/121 (28.1)	11/90 (12.2)	7/90 (7.8)	26/90 (28.9)	30/90 (33.3)

#### Which Digital Services Were Used During the Pandemic?

Three-quarters (114/152, 75%) of the respondents identified (from a predefined list) which digital support services they used during the pandemic. Email (58/114, 50.9%), Zoom or Microsoft Teams (32/114, 28.1%), and WhatsApp, SMS text messaging, or video (10/114, 8.8%) were most commonly used, mirroring data presented in Figure 2. No respondent had used Skype or FaceTime. The most valued digital support services were carers’ well-being assessments, support needs checks, and peer support groups. When asked to indicate which web-based group activities they had experienced (respondents could tick as many as appropriate), 28.8% (34/118) replied. Those carers who provided a response most frequently accessed web-based training and resilience courses (14/24, 58%), virtual yoga sessions or quizzes (10/23, 43%), virtual cafes (9/22, 40%), and “carers evening chat” (4/16, 25%).

A total of 102 comments were entered by participants. These were analyzed for thematic content and commonalities. Three superordinate themes were identified (Textbox 1). These included 8 subthemes (2-3 subthemes emerged for each superordinate theme). A selection of comments relating to each subtheme were extracted. The first theme, how to help carers use digital services in the future, highlighted aspects such as a need for more publicity, activities provided at different times and in different formats, and technical help for persons who are digitally excluded. The second theme, offering a selection of well-being services, contained 2 strong subthemes. One was the view that digital services are invaluable and the second was that face-to-face services are essential for certain functions and for those who are digitally excluded because of their age or for financial reasons. The third and final theme emerging from users was the need to tailor future digital services to meet individual caregivers’ needs. This might include, for example, not only addressing the practical elements of caring or issues of isolation and confidentiality but also acknowledging that “people need more than their problems fixing” and not losing the personal service previously provided, which is highly valued.

**Themes from qualitative analysis of responses.**

**Theme 1: things to help me use digital services in future**

- The need for more publicity and better communications
- “I was not aware of the online services, more information/publicity would be helpful.”
- “Better information of services you can use.”
- “Old fashioned ‘come join us’ flyer through the post. My elderly parents need constant encouragement and they don’t read emails and my repeating them...or reading them out does not have the same impact.”
- Technical help and digital inclusion
- “[C]an’t use a computer, [need] help to set up Zoom.”
- “I am not comfortable using online services.”
- “Not everyone is online so cannot avail themselves to online forums...also the cost of broadband needed to use Zoom etc. which requires higher speed etc. is quite prohibitive if you are living on a fixed income.”
- Activities at different times and in different formats
- “They just don’t fit in as they are at times when I’m caring for my mother.”
- “Due to work pattern [I am] not always available.”
- “A wider range of subjects for the online service. Perhaps short podcasts of interesting places in the world e.g. videos of museums around the world or tourist destinations or cultures and traditions or other countries.”
- “A blend of online and face-to-face better. Also, a brief catch-up call if you can’t make a session as guilt can set in for me if I’m overloaded and I feel unable to continue.”

**Theme 2: offering a selection of well-being services, including on the web**

- Web-based services are invaluable
- “Online saves time travelling and you can access it whilst still caring for the patient in your own home.”
- “Living through COVID has been like being relocated to the moon, no contact with anyone. At a time when in person is still beset with logistical problems the online equivalent is a lifeline.”
- Face-to-face services still necessary
- “It (digital) was a necessary substitute during lockdown but nothing replaces face-to-face interaction.”
- “Nothing is as helpful as face-to-face help, especially where counselling and support services are concerned. Many carers, especially those caring for someone with dementia are elderly and not used to computers.”

**Theme 3: tailoring future services**

- Practical elements of caring
- “A list of possible areas to look at and their contact details e.g. home cleaners, meals on wheels etc.”
- “I have as a parent, many worries about what will happen when I can’t ‘go on’...Legal advice for preparations for the outcome.”
- “Recommended places approved by members experiences. Where to get...Equipment, grants assistance etc. positive recommendations for work carried out for adaptations by local companies.”
- Addressing isolation and confidentiality
- “I feel very isolated as a carer and being able to go to meetings/events/social gathering and see and speak to people normally I feel is very important, both for me as a carer, and my husband who has mixed dementia.”
- “I feel it is of great importance that you can discuss on a one-to-one level in person or telephone on the day. Not every carer can talk freely about what is going on for themselves and especially if the cared for is listening.”
- Addressing changing times and loss of personal service
- “Go back 15 years and the carers center was a place where you could turn up to have a chat with whoever was on the desk. With the move to the library the feel changed— interactions more like ‘please state the nature of your problem’ than ‘how are you, how are things going?’ People need more than their problems fixing...it is more the emotional and community support. That’s it—emotional support as well as practical support.”

## Discussion

### Principal Findings

To our knowledge, this is the first large-scale study to analyze the impact of the COVID-19 pandemic on the provision of well-being services for unpaid caregivers. The longitudinal analysis of >20,000 rural and city carers identified, as expected, a move away from face-to-face to web-based service access. The shift observed mirrors those reported by researchers for other services during the pandemic, such as GP practices [2]. However, over the period from January 2019 to June 2021, the number of monthly contacts handled by the carer support organization more than tripled, with no significant changes in staffing levels. Before the pandemic, digital contacts were exclusively by email or SMS text messaging. During the pandemic, additional options were introduced, including Zoom, WhatsApp, and Microsoft Teams, with new web-based group sessions also offered to carers.

Within the context of a 37% increase in the number of carers registered, the organization managed to increase the number of one-to-one contacts by 88.8%. This increase was particularly evident in rural areas, with the ratio of such carers on the register rising from 1 in 10 carers to 1 in 4 carers by the end of the observation period. Rural carers demonstrated slightly different behaviors, showing much slower initial digital adoption rates. In terms of carers’ rating of the service received, 6 of the 8 quality indicators showed an improvement, and the other two showed only a minor, nonsignificant decrease. The largest improvement was observed in reduced stress, consistent with the findings of a systematic review of caregiver web-based interventions [23]. Our survey identified a high level of computer proficiency among carers, at or above that reported for other older populations [20,22]. Even so, respondents expressed a preference for the organization to continue to offer face-to-face services as well as web-based options to meet a carer’s preferences and the type of well-being support required.

### Comparison With Prior Work

Before the pandemic, researchers reported that the uptake of web-based services by older adults in the United Kingdom remained relatively low, despite their potential benefits [24]. A European examination of web-based services available to support informal carers also found a lack of reliability and usability [25]. A qualitative study of the views of caregivers on suitable technologies to assist their caregiving identified similar themes to this larger study, in particular that digital technology needs to be tailored to users’ needs in order to ensure adoption [26]. Although it is acknowledged that there may be a huge potential to use such tools to support unpaid carers, it is recognized that wholesale adoption may risk inadvertently exacerbating existing support through digital exclusion [27]; for example, the testing of digital tools in a real-world setting has identified a *digital inverse care law*, with those most in need of support least likely to engage with digital health platforms [28]. In addition, a review of eHealth interventions to support caregivers of older adults also highlights the importance of using appropriate language and text, as well as helping caregivers learn how to use the intervention [29].

Our research shows that, during a crisis such as the COVID-19 pandemic, an organization providing support for the well-being of caregivers was able to successfully implement remote service provision using a mix of traditional and digital tools without a detrimental impact on the reported quality of individual contacts and in the context of an increased workload. Systematic reviews of internet-based interventions to support caregivers have to date reported mixed results and called for more high-quality studies [30,31]. A recent review of factors influencing the implementation of eHealth to support informal care found a gap in knowledge regarding success factors and limited focus on the well-being of the unpaid carer, with the focus being principally on the person receiving care [32]. Similarly, studies of telecare that focus on conditions such as dementia usually do not differentiate the caregivers’ needs, instead usually considering the caregiver and the older person or care recipient as a dyad [33-35]. Early in the pandemic, there were some calls to move “carers from the back of the queue” when considering digital services [36]. However, a recent research study of digital interventions for carers of people with dementia still considers need in terms of the dyad, with caregivers in a secondary role [37]. In the United Kingdom, the 2019 report for government on preparing the health care workforce for the digital future recommended that the NHS should work with carer organizations to prioritize the education of patients and caregivers alongside the health care workforce [38].

It is important to consider the indicator that showed a significant deterioration in our study. This was associated with support in accessing health care and social care services, both presumably disrupted by the COVID-19 pandemic. A review of carer support has identified that the ability to coordinate access to such services is particularly valuable, with the integration of home care and community care able to improve outcomes for older people [39,40]. This seems to be particularly important for carers of people with a mental health condition [41]. In Australia, the integration of digital care and clinical care is being assessed to coordinate mental health teams, caregivers, and service users as active partners [42]. The potential for appropriate digital technology to provide support and reassurance is recognized as a benefit for both the caregiver and the person for whom they care [43]. In some parts of the world, volunteers are also being integrated into care to help caregivers use custom-built apps [44].

Investment in innovation to provide optimum support services for informal caregivers could be highly cost-effective. The workforce of unpaid carers represents approximately 6% of the UK population and, together with the 1.3 million registered carers who receive a small carer’s allowance, informal caregivers are widely acknowledged as a crucial component in care delivery [12,45]. Furthermore, since the start of the pandemic, the value of unpaid care provided in England is estimated to be £111 billion (US$ 152.7 billion) [46] and in the United States >US $450 billion annually [47]. The UK government has recently set out a range of policies aimed at empowering unpaid carers, with a dedicated, although small, £25 million (US $26.72 million) budget for this purpose [46]. The danger is that the initiatives will once again focus on caregivers rather the sector that supports them. Thus, the opportunity to integrate organizations such as the one in this study into the wider community-based care system will be missed. This study also questions the stereotype of low digital capability among older carers, with CPQ-12 scores demonstrating high computer proficiency. Even so, survey responses indicate that large-scale naïve digital transformation is unlikely to be effective. Instead of a “one-size-fits-all” approach, there is a need for person-centered support (face-to-face as well as web-based options) as part of the service, together with training for those who need it. Meanwhile, there are emerging indications of a move toward providing solely web-based support, with some suppliers looking ahead to younger and more digitally engaged carers who are assumed not to require face-to-face services [48]. The lower cost of web-based support services may make this seem an attractive option for commissioners in the United Kingdom. A similar situation has occurred in primary care with disruptive innovators entering the NHS market to provide web-based GP services, with the evaluation reporting mixed findings and providers withdrawing from some NHS contracts [49]. For any caregiver support service evaluation, as well as delivery costs, there will need to be a careful consideration of utility (ie, quality and effectiveness from the user perspective) [50]. Although this study was set in England, the findings will be relevant for other countries where digital services to support the well-being of informal caregivers are in use or are being developed.

### Limitations

There are a number of limitations to this study that need to be acknowledged. First, it is unclear how representative, in terms of its digital readiness, the organization studied is of the whole sector. There are no national audits of such carer support organizations, although it is known that >60% of care homes still use internet connections that will not support full digital transformation [16]. Second, the cohort excluded young carers, which inevitably limits generalizability to the wider population of carers [51]. In addition, an important subgroup (working caregivers) could not be identified due to limitations in the data. A quarter of older workers in England currently have caring responsibilities, and this percentage is expected to increase as the population ages [52]. Third and last, although we identified a high level of computer proficiency in survey respondents, this may not be fully representative because most of the respondents (136/152, 89.5%) completed the survey on the web. Further research is needed to provide evidence on these subgroups before drawing any final conclusions about web-based support services.

### Conclusions

Looking to the future, the integration of health care and care services to meet the complex care needs of a country’s aging population is recognized as a global challenge [53]. Considering the importance of unpaid carers, more attention needs to be given in all national strategies to organizations that support this important *free* workforce. Our study highlights a number of issues worthy of further consideration and study that have implications for the design of future cost-effective digital initiatives. These include the lack of any audits of the digital readiness of organizations that provide support for caregivers; the need for a better understanding of rural carers; evidence of the cost-effectiveness as well as the use of different forms of support for caregivers; and the potential for collaboration among different partners within ICSs to better support unpaid caregivers, enhancing their access to, and engagement with, support services after the COVID-19 pandemic.

## Appendix

Multimedia Appendix 1 Metrics and outcomes recorded for adult carers registered with the well-being service.

Multimedia Appendix 2 Caregivers’ feedback questionnaire.

Multimedia Appendix 3 Computer Proficiency Questionnaire, 12-item.

## Abbreviations

CPQ-12: Computer Proficiency Questionnaire, 12-item version
CTHE: Carers Trust Heart of England
GP: general practitioner
ICS: integrated care system
NHS: National Health Service
