# Supplementary material for: Digital Adoption by an Organization Supporting Informal Caregivers During COVID-19 Pandemic Showing Impact on Service Use, Organizational Performance, and Carers’ Well-Being: Retrospective Population-Based Database Study With Embedded User Survey
Source: JMIR Aging. 2024 May 13;7:e46414. doi: 10.2196/46414 (PMC11130774; doi:10.2196/46414)
Supplement: Multimedia Appendix 1 [file aging_v7i1e46414_app1.docx]

**Multimedia Appendix 1: Metrics and outcomes recorded for adult carers registered with the well-being service**

Following discussion with the Performance and Monitoring Officer, a list of routinely recorded carer characteristics and an annotated Summary of Contact Types was agreed for download as shown below. Contact methods included face-to-face, person to person (phone and digital media) and other contact means such as letters.

**Table 1: Routine metrics and outcomes for adult carers registered with the service.**

| **ITEMS DOWNLOADED FOR ANALYSIS** | |
| --- | --- |
| **Data on Registered Adult Carers** | **Contact Method (contd.)** |
| Age | Group Activities |
| Female/male | Home Visit |
| Ethnicity, language needs | Hospital (6 sites) |
| Employed/ unemployed, retired | Outreach (8 named sites) |
| Location (city/rural) | Phone call |
| Carer Assessments | Text |
| Carer Wellbeing checks | Email |
| Referral source (self or directed) | Microsoft Teams |
| Relationship with person cared-for | Zoom |
| Conditions in cared for (e.g. dementia) | Skype / Facetime |
|  | WhatsApp – Text/Video/Call |
| **Number of Contact** | Facebook |
| Annual/ Monthly total number contacts | Letter |
| Per month number contacts |  |
| Annual/ Monthly breakdown by type of contact* | **Benefit Reported Following Contact** |
| Number of one to one contacts with carers | Helped me feel less alone in my caring role |
| Number of carers supported on one-to-one basis | Helped me reduce my stress |
| Number of carer assessments completed | Made my caring role easier to cope with |
| Number of carers attending group activities | Helped me improve my physical health |
|  | Helped me to improve my financial position |
| **Contact Method** | Helped deal with Health & Social Care Professionals |
| Carers Appointment Centre | Helped me increase control of my personal life |
| Carers Centre – Drop in | Helped me increase my confidence |
