# Supplementary material for: Digital Adoption by an Organization Supporting Informal Caregivers During COVID-19 Pandemic Showing Impact on Service Use, Organizational Performance, and Carers’ Well-Being: Retrospective Population-Based Database Study With Embedded User Survey
Source: JMIR Aging. 2024 May 13;7:e46414. doi: 10.2196/46414 (PMC11130774; doi:10.2196/46414)
Supplement: Multimedia Appendix 2 [file aging_v7i1e46414_app2.pdf]

Multimedia Appendix 2: Caregivers Feedback Questionnaire

CARERS TRUST
Heart of England

Supporting Family Caregivers

Background

As a result of COVID-19, we have started to provide a number of our Wellbeing Services (caregivers support) online for carers. Previously, these services were mainly provided through a mix of face-to-face and telephone support.

We now want to ask our clients (adult caregivers) to help us decide which we should plan to provide as a blended service in future (i.e. offering caregivers a choice of online or original style provision). We also want your ideas on what might help us improve provision of these services. Finally, we want to identify other key areas into which we could extend our online and digital services.

Survey Process

We need you to describe your experiences of our Wellbeing Services before and since the COVID-19 pandemic. It is an opportunity for you to tell us anything that you think is relevant.

We would be very grateful if you can help us and fill in this survey by Monday August 16th, answering all the questions applicable to you and giving as many details as possible. It should take about 30 minutes to complete.

Because the questionnaire is intended to cover a wide range of personal circumstances, some of the questions may seem less relevant to you as an individual, but please do persevere. The questionnaire will allow you to skip any questions that you cannot answer.

Please feel free to give any further information or feedback at the end of the survey.

I would like to thank you very much for your help. Your support is very much appreciated and the more caregivers who reply, the more your opinions and experiences will be listened to and acted upon.

Kindest regards

About Your Caring Role

Q1a Your connection to the adult(s) you care for?

- ◯ I am a husband/wife/partner
- ◯ I am a son/daughter
- ◯ I am a friend/neighbour
- ◯ Another connection *(Please Specify…………………………………….)*

Q1b How many years' experience of caring do you have?

[ ] Enter number

Q1c Are the person(s) you care for living on their own?

- ◯ Yes
- ◯ No (If No, please go to **Q1e**)

Q1d If they live on their own, how often do you visit?

- ◯ 1 – 3 times a week
- ◯ 4 – 5 times a week
- ◯ Daily
- ◯ Daily and at Night

Q1e Is additional care provided by a domiciliary 'home care' agency?

- ◯ No
- ◯ Yes

If Yes, how many hours per week [ ] Enter number

Q1f How many hours can the person(s) you care for be left unattended?

[ ] Enter number of hours

Q1g Do you have any school age children?

- ◯ No
- ◯ Yes

Before COVID19 - Your Experience of Our Services

Q2a During 2019, how often did you use the following services?

[please enter one tick ✓ for each service]

**Service**	**How Often Used**		
	Regularly (6-12 times)	Rarely (0-5 times)	Not Used
Carers Centre (Drop in)			
Carers Centre (Appointment)			
Home Visits			
Outreach (e.g. community site)			
Telephone Support			
Group Activities (e.g. peer-to-peer support meetings)			

Q2b If you did not use any of the following services, please explain why.

[please enter one main reason for any service you did not use]

**Service**	**If Not Used Main Reason Why****
Carers Centre (Drop in)	
Carers Centre (Appointment)	
Home Visits	
Outreach (e.g. community site)	
Telephone Support	
Group Activities (e.g. peer-to-peer support meetings)	

** Reason Why :

A = Not needed
B = Not aware of service
C = Not available at times that I needed it
D = Too far to travel
E = Other (please describe)

Q2c Please tell us which of our services was most useful to you and why.

**<u>Most</u> useful service – please describe why**

During Pandemic - Your Experience of Our Services

Q3a During the pandemic, which methods did you use to access our services?

[please enter one tick ✓ for each method provided to access our services]

Method	Used during pandemic	
	Yes	No
Zoom/Microsoft Teams		
Skype / Facetime		
WhatsApp – Text/ Video/ Call		
Email		

Q3b Did you STOP or START using any of our original services below?

[please enter one tick ✓ for each service you stopped or started using]

Service	Frequency of Use		
	Stopped	Started	No effect
Carers Centre (Drop in)			
Home Visits			
Outreach (e.g. community site)			
Telephone Support			

Q3c Did you join any new <u>online or 'virtual'</u> activities introduced for our carers?

○ No (If None, please go to **Q3e**)

○ Yes (If Yes, please tick ✓ for any used; please add others joined)

Activity	Used during pandemic	
	Yes	No
1. Virtual cafes e.g. Carers Virtual Cuppa (Tea@3)		
2. Carers Evening Chat (Virtual Group/ Zoom)		
3. Yoga sessions/ Quiz		
4. Online training and resilience courses		
5. Carers Week events (2020)		
6. Other (please describe……………………….)		
7. Other (please describe……………………….)		
8. Other (please describe……………………….)		

Q3d Which of our various virtual/online services has been most useful to you during the last 16 months and why?

<u>Most</u> useful online service – please explain briefly why

Q3e If you did not use any online service, could anything be done to help you use these in future?

Changes that would help me use an online service– please describe

Your Thoughts on Our Future Service for Caregivers

In a recent "Road Map" survey we carried out, carers suggested activities we should **continue** to offer online **alongside** face-to-face or telephone delivery.

Q4a How important is it for you that we also offer these services online?
[please enter importance level: 5=very important to 0=not important)

**Continue to Offer Online Option..**	**Importance** (0-5)
..For Me as a Caregiver	
1. Carers Assessment/Wellbeing Checks	
2. Meetings about ongoing support needs	
3. Carers clinics for a local area	
4. Virtual training activity (with support)	
5. Peer support groups	
..For Person Cared For *(Indirect support for me)*	
6. Online activities with someone else (e.g. games)	

Q4b If you rated any online service as 0 (Not valuable) or 5 (Very valuable) to continue to offer (alongside face-to-face services), please explain why?

Rated 0 – please identify service & describe briefly why.

Rated 5 – please identify service & describe briefly why.

Q4c Could we provide any further services online to support your caregiving?

Please describe what & briefly explain why

If you have any additional comments, we would be very pleased to hear them.

Thank you for taking the time to complete our survey.

CARERS TRUST
Heart of England
