# Supplementary material for: Digital Adoption by an Organization Supporting Informal Caregivers During COVID-19 Pandemic Showing Impact on Service Use, Organizational Performance, and Carers’ Well-Being: Retrospective Population-Based Database Study With Embedded User Survey
Source: JMIR Aging. 2024 May 13;7:e46414. doi: 10.2196/46414 (PMC11130774; doi:10.2196/46414)
Supplement: Multimedia Appendix 3 [file aging_v7i1e46414_app3.docx]

**Multimedia Appendix 3: Computer Proficiency Questionnaire, 12-item**

**Overview**

The CPQ-12 was chosen because it has been validated in elderly populations, including for hybrid face-to-face and online service models.(20,22) All 152 survey respondents were asked if they could use a computer to access online services; 102 provided a response. Of these, 91% (N =93) said that they could. Of the remainder, 45% (N = 5) said that they would undertake training if it was free, 54% (N = 6) said they would not. Reasons given included, not having a computer or internet access, a dislike of using computers and feeling too old to start learning about computers.

In addition, 94 respondents also completed the CPQ-12 questionnaire. Analysis of responses shows a relatively high score 25.61 (SD 4.40). The questionnaire assesses six types of competence. Basic tasks include using a mouse and keyboard; loading ink into a printer or fixing the printer when the paper jams; using email to communicate, including the ability to open and send emails; and using the internet to find out information about local community resources and to find out about services, hobbies and interests. Confidence was high in these four areas. Respondents were less confident in the two areas covering use of a computer to manage their calendar, enter events, and check the date and time of appointments; and for listening to music and watching films.

Analysis by geographical location (for 84 who provided their location) identified similar levels of computer proficiency for City respondents and Rural carers. Skills levels in the sample were higher than those recorded in the original population which validated the questionnaire in 2015,(20) but similar to those recorded in a more recent 2020 study.(22)

**Table 1: Breakdown Respondents’ Basic Computer Skills Levels (CPQ-12 Questionnaire)**

| **CPQ-12** | **Total group**  **n = 94** | *City postcode*  *Sub-group n = 33* | *Rural postcode*  *Sub-group n = 51* |
| --- | --- | --- | --- |
|  | Mean (SD) | Mean (SD) | Mean (SD) |
| CPQ-12 total | 25.61 (4.40) | 25.18 (4.47) | 26.10 (4.13) |
| 1. Computer basics | 4.59 (0.75) | 4.67 (0.46) | 4.56 (0.83) |
| 2. Printer | 4.18 (1.08) | 3.98 (1.16) | 4.34 (0.89) |
| 3. Communication | 4.84 (0.36) | 4.77 (0.41) | 4.88 (0.32) |
| 4. Internet | 4.37 (0.91) | 4.22 (0.95) | 4.46 (0.84) |
| 5. Calendar | 3.81 (1.42) | 3.79 (1.35) | 3.84 (1.49) |
| 6. Entertainment | 3.89 (1.38) | 3.74 (1.40) | 3.98 (1.39) |

20. Boot WR, Charness N, Czaja SJ, Sharit J, Rogers WA, Fisk AD, et al. Computer proficiency questionnaire: assessing low and high computer proficient seniors. Gerontologist. 2015;55(3):404-11

22. Ratanjee-Vanmali H, Swanepoel W, Laplante-Levesque A. Digital Proficiency Is Not a Significant Barrier for Taking Up Hearing Services With a Hybrid Online and Face-to-Face Model. Am J Audiol. 2020;29(4):785-808
